# Mechanisms of EMT in the immune microenvironment of plasma cell mastitis

**DOI:** 10.3389/fimmu.2025.1637725

**Published:** 2025-09-12

**Authors:** Meng Zhou, Yubi Zhang, Yuanhao Shao, Bin Wu, Jing Zhou

**Affiliations:** ^1^ Department of Thyroid and Breast Surgery, Union Hospital, Tongji Medical College, Huazhong University of Science and Technology, Wuhan, China; ^2^ Department of Orthopedics, Union Hospital, Tongji Medical College, Huazhong University of Science and Technology, Wuhan, China; ^3^ Department of Thyroid and Breast Surgery, People’s Hospital of Dongxihu District Wuhan City and Union Dongxihu Hospital, Huazhong University of Science and Technology, Wuhan, China

**Keywords:** plasma cell mastitis (PCM), epithelial mesenchymal transition (EMT), fibrosis, immune microenvironment, inflammatory cytokines, pattern recognition receptors

## Abstract

Plasma cell mastitis (PCM), a prevalent and refractory form of non-lactating mastitis, is characterized by the pathological triad of ductal ectasia (DE), plasma cell-dominated inflammatory infiltration, and progressive fibrosis. Despite its clinical burden, current surgical interventions yield suboptimal outcomes with recurrence rates up to 43%, underscoring an urgent need for mechanistic insights. This review synthesizes evidence establishing epithelial-mesenchymal transition (EMT) as a central driver of PCM pathogenesis, intricately regulated by the disease-specific immune microenvironment. We demonstrate that autoimmune-mediated DE initiates ductal damage, generating damage-associated molecular patterns (DAMPs) that activate pattern recognition receptors (PRRs). This triggers NF-κB signaling hubs, upregulating pro-inflammatory mediators (IL-1β, IL-6, TGF-β1, ICAM-1, CXCL12) and core EMT-transcription factors (Snail, TWIST). Crucially, IL-6/JAK/STAT3 signaling promotes plasma cell survival via Bcl-2 while concurrently driving EMT in ductal epithelium. Concurrently, IL-1βactivate PI3K/Akt to stabilize EMT effectors and enhance ECM synthesis. A unique, self-amplifying “EMT-fibrosis loop” is identified as a PCM hallmark: EMT-derived fibroblasts secrete CXCL12 and TGF-β1, which activate NF-κB pathways in adjacent epithelia to perpetuate EMT and ECM deposition. This loop, alongside sustained plasma cell activity via IL-6/STAT3/Bcl-2, underpins PCM’s chronicity and distinguishes it from other mastitides like granulomatous lobular mastitis (GLM). We further highlight exosomal involvement in CXCL12 transport and M1 macrophage polarization as amplifiers of inflammation and EMT. Targeting these convergent pathways (NF-κB, JAK/STAT3) or disrupting the EMT-fibrosis loop (e.g., via CXCL12/TGF-β1 inhibitors) represents a promising therapeutic strategy to mitigate fibrosis and recurrence. Future research must validate these mechanisms in human-relevant models and address critical gaps in bacterial-autoimmune interplay and temporal dynamics across PCM stages.

## 1 Introduction

PCM represents a prevalent chronic inflammatory disorder of mammary tissue, predominantly occurring in non-lactating females aged between 30 and 50 years (1). In recent years, the incidence of PCM has been increasing, placing a considerable burden on affected women (2). Clinically, PCM typically presents as a painful, periareolar mass often associated with nipple retraction, skin erythema, and, in advanced stages, fistula formation with persistent discharge (3).

At present, clinical management primarily involves conservative approaches (e.g., antibiotics, anti-inflammatory drugs, drainage) or conventional surgical interventions (e.g., lesion excision, duct excision, mastectomy) (4, 5). Conservative treatments, although less invasive, are frequently considered ineffective in halting disease progression or preventing recurrence in many patients, leading to prolonged suffering and repeated interventions (6). Therefore, most scholars continue to advocate for surgical excision. However, the scars and breast deformity left by surgery significantly affect the appearance of the breasts (7), and the postoperative recurrence rate is as high as 43% (4). The severe clinical symptoms and prolonged course of the disease significantly impact patients’ quality of life. This therapeutic dilemma underscores the urgent need for a deeper understanding of PCM pathogenesis to develop more effective and less morbid treatment strategies.

The initial manifestation of PCM is dilation of the mammary ducts (3). The lumens of these affected ducts are filled with secretions, exfoliated epithelial cells, and foamy histiocytes, showing notable dilation (8). The precise triggers initiating DE remain incompletely elucidated, but several hypotheses exist. Autoimmune mechanisms are strongly implicated, as evidenced by significantly elevated levels of antinuclear and antihistone antibodies in PCM patients compared to healthy controls (9). This is further supported by the effectiveness of glucocorticoid and immunosuppressive therapies (10, 11). Additionally, ductal obstruction leading to secretory stasis, potential roles of subclinical bacterial infection or dysbiosis altering the local microenvironment, and hormonal influences (e.g., hyperprolactinemia) have also been proposed as contributing factors to DE initiation (12). Following DE, the duct walls undergo progressive thickening and fibrotic changes, concurrent with atrophy of the epithelial lining. Over time, a marked diffuse infiltration of lymphocytes, plasma cells, and additional inflammatory mediators surrounds the ducts, with plasma cells exhibiting particularly pronounced accumulation (13). This persistent inflammatory milieu drives progressive fibrosis, a defining pathological feature of PCM that contributes significantly to tissue damage and therapeutic resistance.

A central cellular process implicated in the development of fibrosis across various organs, including potentially the breast in chronic inflammatory settings like PCM, is the epithelial-mesenchymal transition (EMT) (14). The EMT is a dynamic biological mechanism through which epithelial cells undergo phenotypic conversion into mesenchymal cells. This transformation facilitates the generation of fibroblasts and myofibroblasts, which actively produce and secrete extracellular matrix (ECM) constituents, thereby contributing to ECM remodeling (15). The pathological deposition of extracellular matrix (ECM) components, driven by persistent inflammatory stimuli, can lead to fibrotic tissue formation. During this process, fibroblasts serve as key effector cells through their secretion of various inflammatory mediators, including cytokines, chemokines, and adhesion molecules, which perpetuate the inflammatory cascade and promote fibrogenesis (16). Thus, the development of chronic inflammation and its fibrotic sequelae are significantly modulated by EMT (17, 18). Molecularly, EMT is characterized by the downregulation of epithelial markers (e.g., E-cadherin) and the upregulation of mesenchymal markers (e.g., vimentin, N-cadherin, fibronectin). This phenotypic shift is orchestrated by core transcriptional regulators, including SNAIL, TWIST, and zinc-finger E-box-binding (ZEB) proteins, which coordinately drive the conversion from epithelial to mesenchymal cellular states (19, 20).

Supporting the relevance of EMT to PCM, ultrastructural studies using transmission electron microscopy have revealed compelling evidence. Studies found that individuals with DE have vimentin-positive cells within their normal epithelial cells. The cytoplasm of these epithelial cells contained fibril-like material, and their nuclei were elongated (21). Additionally, PCM was found to have a disrupted ductal epithelial basement membrane, with fewer gap junctions and mosaic connections. Furthermore, an increased presence of lipid droplets, microfilaments, free ribosomes, and cytoplasmic lumens was observed between neighboring membranes (22). These morphological alterations strongly suggest that mammary epithelial cells in PCM undergo EMT. This transition is hypothesized to facilitate the extensive ECM remodeling observed, which may ultimately promote the development of breast fibrosis and the propagation of inflammation within the gland.

Therefore, this review comprehensively examines the process of EMT in PCM mammary epithelial cells, with particular emphasis on its intricate regulation by components of the unique immune microenvironment characteristic of this disease. By examining the interplay between immune microenvironment dynamics and fibrotic progression in mammary tissue, we elucidate pathogenic mechanisms underlying PCM. These insights may facilitate the development of innovative diagnostic approaches and targeted therapeutic strategies for this challenging breast disorder.

To clarify the molecular players involved in PCM pathogenesis, key terms related to inflammatory mediators are defined as follows (**Table 1**):

**Table 1:** Summary table.

Inflammatory mediator	Key signaling pathways	Role in PCM-related EMT and fibrosis
IL-1β	MEK/ERK, PI3K/Akt, NF-κB	Activates partial EMT via TWIST and SNAIL; amplifies inflammation
IL-6	JAK/STAT3	Promotes plasma cell survival (via Bcl-2) and EMT (via SNAIL)
TGF-β1	TGF-β/Smad	Induces EMT; drives ECM synthesis; forms positive feedback with EMT-derived fibroblasts
ICAM-1	NF-κB	Enhances immune cell adhesion; amplifies NF-κB-mediated EMT via SNAIL
CXCL12	NF-κB, PI3K/Akt/mTOR	Recruits fibroblasts; induces EMT in epithelial cells; perpetuates “EMT-fibrosis loop”
TLR4	NF-κB	Activated by DAMPs; upregulates pro-inflammatory cytokines and SNAIL to drive EMT
NLRP3	Caspase-1/IL-1β	Promotes IL-1β maturation; indirectly accelerates EMT and fibrosis

Cytokines: Small proteins or peptides secreted by immune and non-immune cells (e.g., epithelial cells, fibroblasts) that regulate immune responses, inflammation, and cell differentiation. In PCM, critical cytokines include interleukin-1β (IL-1β), interleukin-6 (IL-6), and transforming growth factor-β1 (TGF-β1).

Chemokines: A subset of cytokines specifically involved in cell migration, guiding immune cells to sites of inflammation. In PCM, CXC motif ligand 12 (CXCL12) is a key chemokine.

Pattern recognition receptors (PRRs): Cell surface or intracellular receptors that detect pathogen-associated molecular patterns (PAMPs) or damage-associated molecular patterns (DAMPs), initiating innate immune responses. In PCM, PRRs include Toll-like receptor 4 (TLR4), NLRP3 inflammasome, and nucleotide-binding oligomerization domain 2 (NOD2).

Adhesion molecules: Cell surface proteins that mediate cell-cell or cell-ECM interactions, facilitating immune cell recruitment and retention. Intercellular adhesion molecule-1 (ICAM-1) is a critical adhesion molecule in PCM.

Inflammatory mediators: these molecules—along with other soluble factors and signaling intermediates—are referred to as inflammatory mediators, which coordinate the complex interplay between immune dysregulation, EMT, and fibrosis in PCM.

## 2 Methods

The PubMed database was searched for articles published from 1 January 1990 to 1 May 2025 using the following search terms: “plasma cell mastitis”, “periductal mastitis”, “mammary duct ectasia”, and “non-puerperal mastitis”. The results from the identified literatures were analyzed and summarized.

## 3 Result

### 3.1 Inflammatory mediators and EMT in the PCM inflammatory response

#### 3.1.1 IL-1β

**Figure 1:**
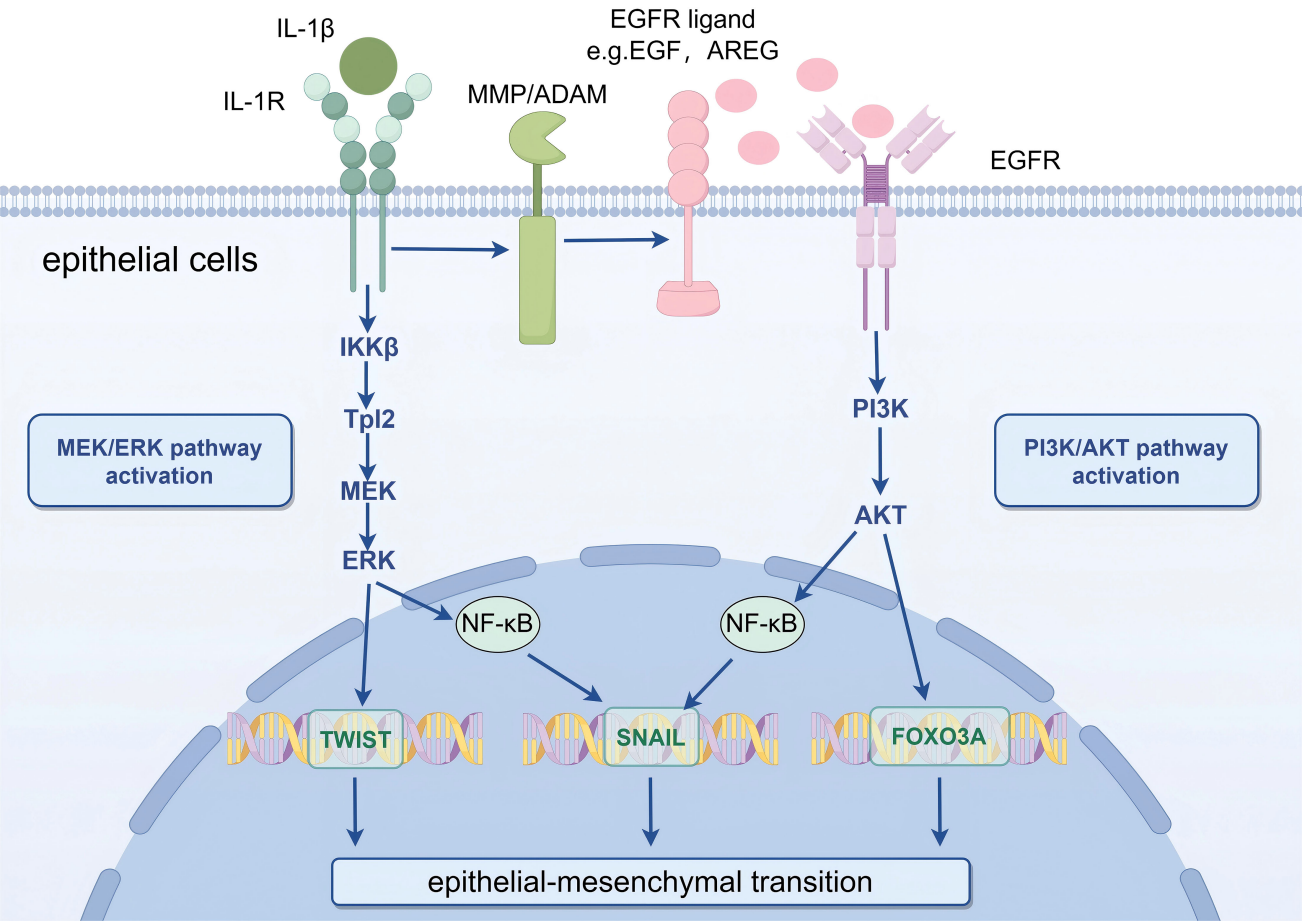
Schematic illustration of IL-1β-mediated partial EMT in mammary epithelial cells via MEK/ERK and PI3K/Akt signaling pathways. Key message: IL-1β acts as a critical mediator to induce partial EMT in PCM by activating two parallel signaling cascades, which synergistically upregulate EMT-related transcription factors. IL-1β, Interleukin-1β; IL-1R, IL-1 Receptor; EGFR, Epidermal Growth Factor Receptor; MMP, Matrix Metalloproteinase; ADAM A Disintegrin and Metalloproteinase; PI3K, Phosphatidylinositol 3-Kinase; Akt, Protein Kinase B; MEK, Mitogen-Activated Protein Kinase Kinase; ERK, Extracellular Signal-Regulated Kinase; NF-κB, Nuclear Factor Kappa-Light-Chain-Enhancer of Activated B Cells; FOXO3A, Forkhead Box O3A. Biological process explanation: IL-1β first binds to its receptor IL-1R on the surface of mammary epithelial cells, triggering two independent signaling pathways. On one hand, the Tpl2-mediated MEK/ERK pathway is activated, leading to ERK phosphorylation; phosphorylated ERK further upregulates the expression of the EMT-transcription factor TWIST. On the other hand, EGFR is activated, initiating the PI3K/Akt pathway; phosphorylated Akt modulates the activity of NF-κB and FOXO3A, and these two regulators coordinately promote the expression of the EMT-transcription factor SNAIL. The combined action of TWIST and SNAIL ultimately drives mammary epithelial cells to undergo partial EMT, which contributes to the fibrotic progression of PCM.

IL-1β stimulates the NF-κB signaling cascade, leading to enhanced expression of intercellular adhesion molecule-1 (ICAM-1) and consequent amplification of localized inflammatory processes (23). Additionally, IL-1β induces part of the EMT process (24). Specifically, IL-1β stimulates the epidermal growth factor receptor (EGFR), thereby initiating the PI3K/Akt signaling cascade (25). The resultant Akt phosphorylation modulates multiple transcriptional regulators, including NF-κB and FOXO3A, which are critically involved in orchestrating EMT (26). Beyond its EGFR-mediated activation of PI3K/Akt signaling, the IL-1 receptor-dependent MEK/ERK pathway is also crucial for IL-1β-induced partial EMT (**Figure 1**) (27). Experimental evidence demonstrates that IL-1β coordinately activates both MEK/ERK and PI3K/Akt signaling cascades, with their synergistic interaction being indispensable for the induction of partial epithelial-mesenchymal transition (28).

In PCM, the expression of IL-1β is elevated (29). KEGG pathway enrichment analysis reveals a significant association between PI3K/Akt signaling and PCM pathogenesis, identifying EGFR as a potential therapeutic target (30). Based on these findings, we hypothesize that IL-1β may promote EMT within the PCM microenvironment through PI3K/Akt pathway activation, subsequently driving ECM remodeling and potentiating fibrotic progression.

#### 3.1.2 IL-6

IL-6 serves as a pivotal inflammatory cytokine that regulates both immune regulation and inflammatory cascades. Prolonged overexpression of IL-6 has been pathologically linked to multiple chronic inflammatory disorders, including rheumatoid arthritis (31) and inflammatory bowel disease (32). Additionally, in the inflammatory microenvironment of gastric cancer and lung adenocarcinoma, IL-6 stimulates the JAK/STAT signaling pathway, regulates the expression of Snail, promoting the EMT process (33, 34). High levels of IL-6 are frequently observed in numerous chronic inflammatory conditions associated with fibrosis. IL-6 mediates fibrotic progression in diverse organ systems primarily through JAK/STAT3 pathway activation, serving as a central regulator of ECM deposition and tissue remodeling (35).

The upregulation of IL-6 is strongly associated with the pathophysiology of mastitis (36),and is found to be significantly elevated in PCM (37). Chen et al. established an experimental PCM model by administering IL-6 into the mammary glands of mice implanted with human breast tissue homogenates. This intervention activated the IL-6/JAK/STAT3 signaling cascade, which recapitulates key pathological features of PCM—including significant upregulation of Bcl-2 expression in plasma cells that closely mirrors clinical observations in human patients (38). Based on these findings, it can be concluded that in PCM, mammary duct epithelial cells secrete IL-6, thereby initiating JAK/STAT3 pathway activation. This stimulation drives B lymphocytes to differentiate into plasma cells, which accumulate around the mammary ducts. Meanwhile, STAT3 also promotes the production of its downstream anti-apoptotic gene, Bcl-2, which ensures the survival of plasma cells. IL-6 additionally enhances JAK/STAT in mammary ductal epithelial cells, and STAT3 upregulates Snail expression, further promoting EMT in PCM mammary ductal epithelial cells.

#### 3.1.3 ICAM-1

ICAM-1 represents a pivotal cell surface glycoprotein predominantly expressed on epithelial cells, serving as a crucial mediator of immune surveillance and inflammatory processes (39, 40). In an inflammatory environment, NF-κB undergoes activation and nuclear translocation, where it transcriptionally upregulates ICAM-1 expression. Meanwhile, ICAM-1 activates the upstream signaling pathway of NF-κB, resulting in a positive feedback loop (41, 42). This feedback mechanism facilitates NF-κB nuclear translocation and subsequent binding to Snail gene promoter elements, thereby potentiating transcriptional activation and promoting EMT progression (43). This positive feedback loop further amplifies ICAM-1 expression within the inflammatory microenvironment.

The upregulation of ICAM-1 may lead to interactions between epithelial cells and the surrounding matrix or immune cells, facilitating the exchange of intercellular signals (44). These interactions not only enhance the occurrence of EMT but may also cause changes in ECM components, thus affecting cell migration and function.

Dong et al. demonstrated significantly elevated NF-κB p65 and ICAM-1 protein expression levels in breast tissue specimens from PCM patients compared to healthy controls. Importantly, their findings revealed a strong positive correlation between NF-κB p65 and ICAM-1 expression levels, suggesting potential functional interplay in PCM pathogenesis (45). Considering that PCM is an inflammatory condition secondary to mammary duct dilation, it can be hypothesized that NF-κB p65 in mature plasma cells may be activated, leading to the overexpression of ICAM-1. This overexpression may drive EMT in breast epithelial cells while also promoting the adherence, extravasation, and aggregation of inflammatory cells around the mammary ducts.

#### 3.1.4 TGF-β1

TGF-β expression is upregulated in response to inflammatory stimuli and tissue injury, playing a pivotal role in promoting fibrotic remodeling across multiple organ systems (46, 47). TGF-β1 is an essential inducer of EMT (48). On the cell surface, it binds to TGF-β receptors, thereby activating its downstream effector molecules, Smad2 and Smad3 (49). Following phosphorylation, these signaling molecules undergo nuclear translocation, where they activate the transcription of EMT-associated genes (50). Simultaneously, the mesenchymal cells generated during the EMT process secrete additional TGF-β1, creating a positive feedback cycle that sustains EMT progression, ECM remodeling, and fibrotic tissue formation.

Chen et al. observed a significant elevation of TGF-β1 in mastitis tissues. Subsequent *in vitro* experiments revealed that TGF-β1 stimulation of normal bovine mammary epithelial cells robustly activated the TGF-β/Smad signaling pathway (51). In a bovine model of mastitis (a system with noted differences from human PCM), this activation triggered EMT in mammary epithelial cells, contributing to increased synthesis of ECM components and subsequent development of mammary fibrotic tissue. Another study suggests that mammary fibroblasts may serve as a key source of TGF-β1 (52). However, it is important to note that bovine mammary physiology and the pathological context of mastitis in cattle differ substantially from those of human PCM. While these findings in bovine mammary cells are suggestive, critical validation is needed to determine whether the same TGF-β1/Smad signaling pathway drives EMT in human periductal epithelial cells during PCM progression. Further *in vivo* studies involving repeated injections of TGF-β1 in mice revealed pronounced fibrosis and inflammatory cell infiltration in the mammary glands, reinforcing the role of TGF-β1 in driving these pathological processes. Nevertheless, the murine model relies on exogenous TGF-β1 administration, which does not fully recapitulate the spontaneous, chronic inflammatory microenvironment of human PCM. The extent to which TGF-β1-induced fibrosis in mice mirrors the progressive, ductal ectasia-associated fibrosis in humans remains to be clarified through studies using human PCM tissues or more clinically relevant models.

Additionally, research on bovine mammary epithelial cell lines has identified the long non-coding RNA H19 as a novel regulator of TGF-β1-induced EMT. In a bovine model of mastitis (a system with noted differences from human PCM), H19 modulates both TGF-β1-induced EMT and the production of ECM components through the PI3K/Akt signaling cascade, thereby promoting the formation of mammary fibrotic tissue (53). Again, the translational relevance of these findings to human PCM is uncertain. Bovine mammary epithelial cells may exhibit distinct regulatory mechanisms of lncRNA-mediated EMT compared to human mammary ductal epithelial cells, particularly in the context of PCM’s unique immune microenvironment. Future studies should prioritize investigating whether H19 or analogous lncRNAs play a conserved role in human PCM-associated EMT and fibrosis.

#### 3.1.5 CXCL12

CXCL12, a small cytokine from the chemokine family, is the sole ligand for the CXCR4, which is predominantly expressed on fibrocytes (54). The CXCL12/CXCR4 chemokine axis serves as a critical mediator of inflammatory processes through two principal mechanisms (1): chemoattraction of immune cells to inflammatory sites, and (2) facilitation of B lymphocyte differentiation into plasma cells (55, 56). Furthermore, the CXCL12/CXCR4 axis promotes fibroblast recruitment to focal zones, as well as their activation, proliferation, migration, and ECM component secretion (57). Current evidence implicates the CXCL12/CXCR4 chemokine axis as a potent mediator of EMT and fibrotic pathogenesis across multiple organ systems. Mechanistic studies have demonstrated its substantial involvement in promoting ECM deposition and tissue remodeling in diverse fibrotic disorders.

He et al. demonstrated that inflammation-associated fibroblasts (INFs) isolated from mastitis-affected cows’ mammary glands exhibit significantly higher mRNA and protein expression levels of CXCL12 compared to normal control tissues, and these INFs can induce epithelial cells to undergo EMT (58). In a bovine model of mastitis (a system with noted differences from human PCM), mechanistically, exogenous CXCL12 treatment of bovine mammary epithelial cells led to a significant increase in p65 phosphorylation, suggesting that CXCL12 may trigger EMT and inhibit epithelial cell proliferation through the NF-κB pathway. Furthermore, experimental evidence demonstrates that exogenous CXCL12 administration induces mastitis pathogenesis in murine models (a system with noted differences from human PCM), characterized by both inflammatory infiltration and interstitial fibrosis, indicating that stromal fibroblast-secreted CXCL12 exacerbates inflammatory responses and promotes epithelial cell EMT via paracrine signaling—a critical pathway for sustained inflammation and progressive fibrotic remodeling in the mammary gland microenvironment.

**Figure 2:**
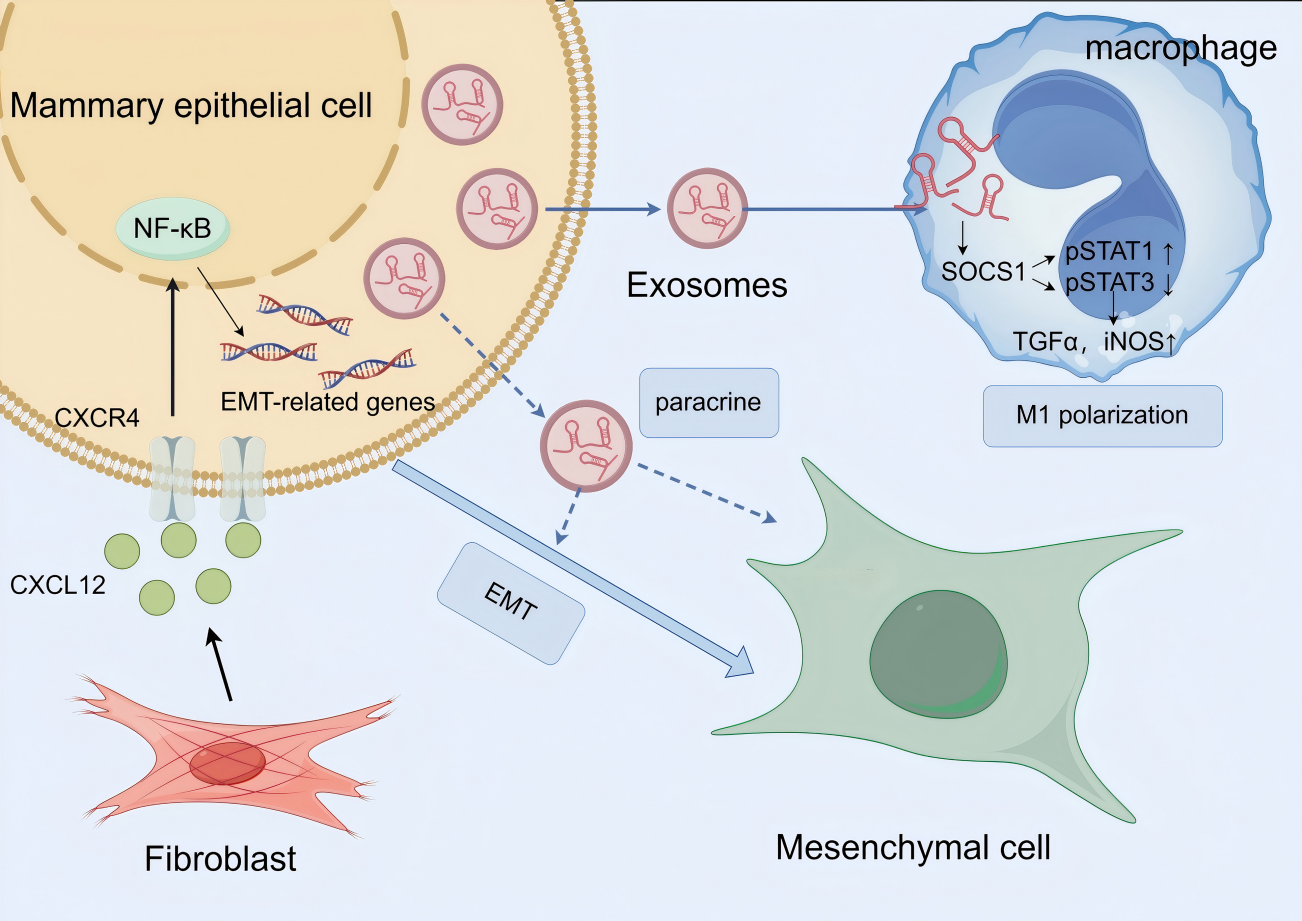
Schematic diagram of exosome involvement in macrophage M1 polarization and epithelial-mesenchymal transition (EMT) to promote mammary inflammation and fibrosis in PCM. Key message: Exosomes play dual roles in PCM pathogenesis—they participate in CXCL12-mediated EMT induction and drive M1 macrophage polarization to amplify inflammation, ultimately exacerbating mammary tissue fibrosis. NF-κB, Nuclear Factor Kappa-Light-Chain-Enhancer of Activated B Cells; SOCS1, Suppressor of Cytokine Signaling 1; STAT, Signal Transducer and Activator of Transcription; TGF-α, Transforming Growth Factor-α; iNOS, Inducible Nitric Oxide Synthase. Biological process explanation: 1. Fibroblasts in the PCM microenvironment secrete CXCL12, which binds to the CXCR4 receptor on the surface of mammary epithelial cells; this binding activates the NF-κB signaling pathway, upregulates the expression of EMT-related genes, and ultimately induces epithelial cells to undergo EMT, converting them into mesenchymal cells. Exosomes may act as carriers to transport CXCL12, enhancing its paracrine effect on epithelial cells. 2. Mammary epithelial cells secrete exosomes containing miR-221; these exosomes are taken up by macrophages and regulate the SOCS1/STAT signaling pathway (SOCS1 is a target gene of miR221; activation of SOCS1 promotes p-STAT3 and inhibits p-STAT1). This pathway activation promotes macrophage M1 polarization, and polarized M1 macrophages secrete pro-inflammatory mediators such as TGF-α and iNOS, which further exacerbate the local inflammatory response in the mammary gland. The amplified inflammation indirectly promotes EMT progression, forming a positive feedback loop between inflammation and EMT to accelerate PCM-related fibrosis.

Notably, this CXCL12-mediated paracrine effect may be closely linked to exosomal mechanisms (**Figure 2**). Studies have reported that CXCL12 secreted by LTA-stimulated primary mammary fibroblasts contributes to fibrotic progression by triggering EMT in mammary epithelial cells, a process potentially associated with exosomal paracrine effects. In PCM, the role of exosomes is further supported by evidence suggesting their involvement via the PI3K/Akt/mTOR signaling pathways, which may synergize with CXCL12-driven NF-κB activation to amplify EMT (22, 58, 59). Additionally, exosomes secreted by mammary epithelial cells can carry miR-221 through the SOCS1/STATs pathway to stimulate M1 polarization and enhance the inflammatory response, possibly forming a positive feedback loop that further reinforces inflammatory response and EMT. stimulate M1polarization and enhance the inflammatory response in LTA-treated animals via the SOCS1/STATs pathway, potentially creating a feedforward loop that reinforces CXCL12-mediated EMT and inflammation (60).

Together, these findings point to a coordinated network where stromal fibroblast-derived CXCL12, possibly transported or modulated by exosomes, drives EMT through NF-κB and PI3K/Akt/mTOR signaling, contributing to the chronic inflammation and fibrosis characteristic of PCM. However, the precise mechanisms by which CXCL12 interacts with exosomes to promote EMT specifically in PCM remain to be fully elucidated.

### 3.2 PRRs and EMT in the PCM immune response

#### 3.2.1 TLR4

TLR4 serves as a critical pattern recognition receptor that mediates the detection of both PAMPs and DAMPs. This recognition mechanism triggers the activation of innate immune responses (61). In PCM, TLR4 has emerged as a potential target for immunotherapy (62), with its activation known to promote local inflammation and alter cell signaling, ultimately influencing the EMT process. Notably, lipopolysaccharide (LPS)—a major component of the outer membrane of Gram-negative bacteria—has been shown to robustly induce EMT in various cell types through both *in vitro* and *in vivo* models (63). Upon LPS binding to TLR4, TLR4 dimerizes and initiates a MyD88-dependent signaling cascade, which culminates in NF-κB activation and the transcription of pro-inflammatory mediators (61).

Liu et al. observed marked upregulation of core components within the TLR4/NF-κB/Snail signaling axis in mastitis tissues compared to healthy controls, suggesting the potential involvement of this pathway in mastitis pathogenesis (64). Experimental studies demonstrate that combined LPS and TGF-β1 treatment induces EMT in normal goat mammary epithelial cells (GMECs). In a goat model of mastitis (a system with noted differences from human PCM), following LPS stimulation, significant upregulation of both NF-κB p65 and the EMT-transcription factor Snail was observed. Pharmacological inhibition of TLR4/NF-κB signaling using TAK-242 effectively suppressed Snail expression and p65 phosphorylation (p-p65), consequently attenuating LPS-induced EMT in GMECs. Importantly, immunohistochemical analysis revealed elevated TLR4 expression across various stages of PCM, strongly implicating the TLR4/NF-κB/Snail signaling axis in PCM pathogenesis (36). However, critical limitations of these preclinical findings must be acknowledged. The GMEC model, while informative, reflects mammary physiology in goats, which differs substantially from the human mammary ductal microenvironment. It remains unclear whether TLR4-mediated EMT in goat cells translates to human PCM, where the inflammatory milieu and epithelial cell characteristics are distinct. Additionally, the *in vitro* induction of EMT using exogenous LPS and TGF-β1 does not fully recapitulate the chronic, self-sustaining inflammatory cascades in human PCM. Further studies using human PCM tissues or patient-derived epithelial cell models are essential to validate whether the TLR4/NF-κB/Snail pathway plays a conserved role in driving EMT in human disease.

#### 3.2.2 NLRP3

As a key component of the NOD-like receptor family, NLRP3 serves as a critical regulator of apoptotic processes and inflammatory cascades (65, 66). The NLRP3 inflammasome, a multiprotein complex comprising NLRP3, the adaptor protein ASC (apoptosis-associated speck-like protein containing a CARD), and the effector enzyme caspase-1, mediates inflammatory responses through caspase-1 activation. Upon inflammasome assembly in inflammatory microenvironments, activated caspase-1 cleaves pro-IL-1β and pro-IL-18 into their bioactive forms (67). Notably, mature IL-1β induces significant remodeling of ECM components, thereby promoting fibrotic tissue transformation.

According to Sun et al., NLRP3 expression is markedly elevated in a mouse model of PCM (29). Pharmacological inhibition of NLRP3 inflammasome signaling using MCC950 in inflamed mammary tissue demonstrated significant attenuation of both NLRP3 expression and downstream effector molecules, including caspase-1 and mature IL-1β. This effect is achieved by decreasing plasma cell infiltration and enhancing recruitment and immunosuppressive capacity of myeloid-derived suppressor cells (MDSCs). Based on these findings, we hypothesize that NLRP3 contributes to mammary tissue fibrosis and plasma cell infiltration during the inflammatory response in PCM.

#### 3.2.3 NOD2

NOD2 represents an additional cytosolic pattern recognition receptor within the NOD-like receptor family. This receptor specifically interacts with muramyl dipeptide (MDP), a conserved microbial motif present in both Gram-positive and Gram-negative bacterial cell walls (68, 69). Studies have shown that *Fusobacterium nucleatum* can induce neutrophil extracellular traps (NETs) formation via the TLR4-ROS and NOD1/2 signaling pathways in neutrophils, accelerate tumor growth, and promote tumor metastasis, manifested as EMT-associated cell migration (70). NOD2 is essential for detecting pathogenic bacteria in mammary epithelial cells and initiating innate immune responses (71). Studies suggest that PCM is an inflammatory disease associated with an immune response triggered by bacterial infection (2). Mammary epithelial cells utilize NOD2 to detect MDP when the mammary gland is infected by bacteria. This triggers the NF-κB signaling cascade, resulting in the production of pro-inflammatory mediators and chemotactic cytokines (1), and may be associated with the occurrence of EMT in PCM.

## 4 Discussion

PCM, a prevalent and clinically challenging form of non-puerperal mastitis, is characterized by a triad of pathological features: ductal ectasia (DE), plasma cell-dominated inflammation, and progressive fibrosis. Central to its pathogenesis is a self-amplifying circuit that intertwines autoimmune-triggered ductal damage, immune microenvironment dysregulation, and EMT-mediated fibrosis. This integrated network, orchestrated by key inflammatory mediators and signaling pathways, distinguishes PCM from other breast inflammatory conditions and underpins its recalcitrance to current therapies.

### 4.1 The core “EMT-Fibrosis Loop”: a central driver of PCM chronicity

**Figure 3:**
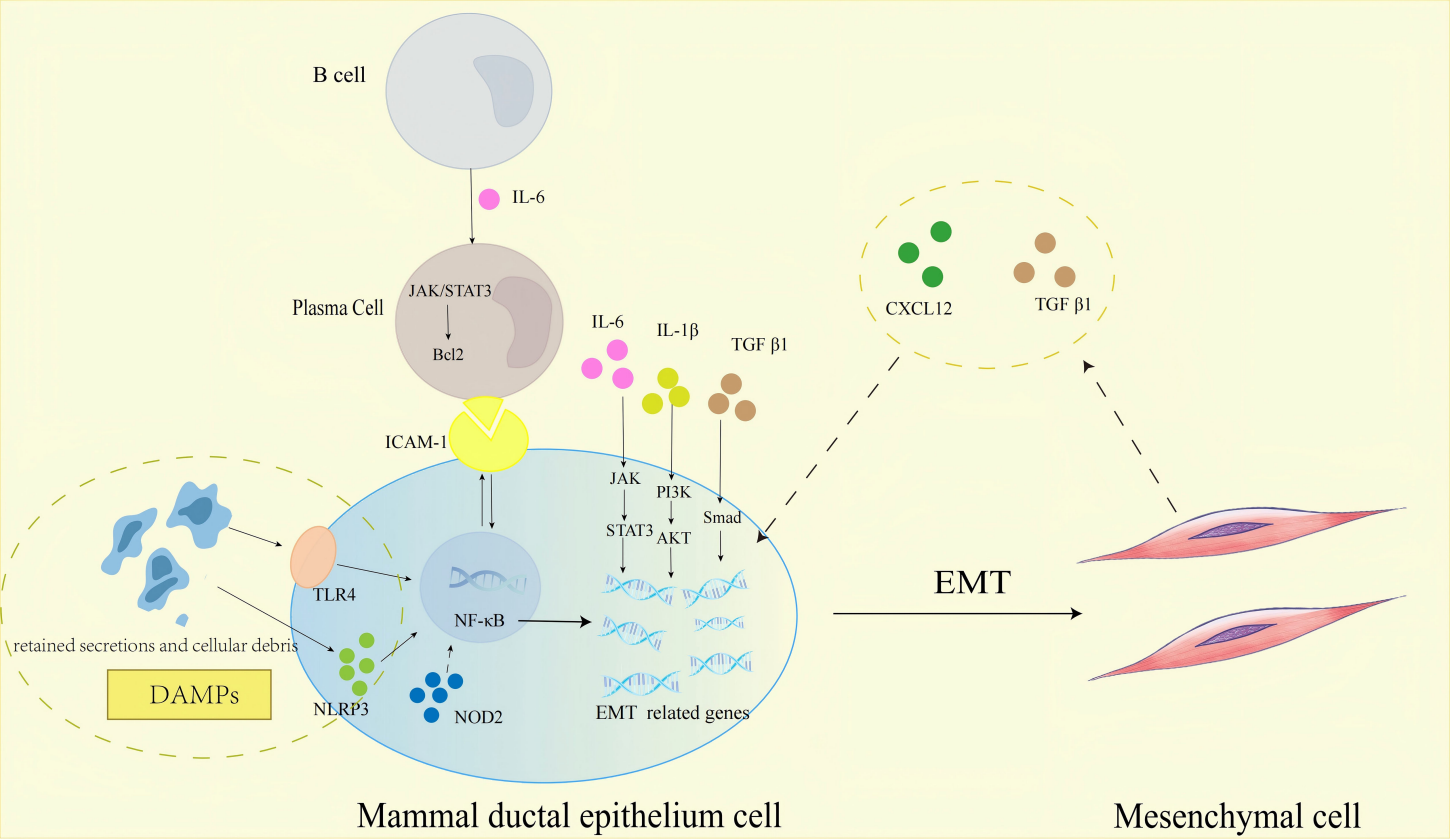
Schematic representation of the molecular pathways regulating EMT in mammary ductal epithelial cells within the PCM immune microenvironment, highlighting the core “EMT-fibrosis loop”. Key message: The self-amplifying “EMT-fibrosis loop”—orchestrated by DAMPs, PRRs, inflammatory mediators, and immune cells—is the central driver of PCM chronicity and fibrosis. JAK, Janus Kinase; STAT3, Signal Transducer and Activator of Transcription 3; PI3K, Phosphatidylinositol 3-Kinase; Akt, Protein Kinase B; Bcl-2, B-Cell Lymphoma 2; NF-κB, Nuclear Factor Kappa-Light-Chain-Enhancer of Activated B Cells. Biological process explanation: 1. Initiation: Autoimmune-mediated DE leads to accumulation of retained secretions and cellular debris in mammary ducts, generating DAMPs. DAMPs activate PRRs (TLR4, NLRP3, NOD2) on mammary ductal epithelial cells, triggering NF-κB signaling. 2. Inflammation and EMT induction: Activated NF-κB drives the transcription of pro-inflammatory mediators (IL-1β, IL-6, ICAM-1). IL-6 binds to receptors on B cells, activating the JAK/STAT3 pathway; this pathway not only promotes B cell differentiation into plasma cells but also upregulates Bcl-2 to enhance plasma cell survival (sustaining the inflammatory infiltrate). Meanwhile, IL-6/JAK/STAT3, IL-1β/PI3K/Akt, and TGF-β1/Smad pathways collectively upregulate EMT-related genes, inducing mammary ductal epithelial cells to undergo EMT. 3. “EMT-fibrosis loop” formation: Epithelial cells undergoing EMT differentiate into mesenchymal cells (fibroblast-like cells), which secrete CXCL12 and TGF-β1. CXCL12 and TGF-β1 act on adjacent mammary ductal epithelial cells, reactivating EMT-related signaling pathways to perpetuate EMT; this forms a self-amplifying loop that drives continuous ECM deposition and mammary tissue fibrosis, maintaining PCM chronicity. Solid arrows denote pathways with direct evidence in PCM; dashed arrows represent proposed interactions in PCM.

At the heart of PCM pathogenesis lies a unique, self-amplifying “EMT-fibrosis loop” that sustains disease progression (**Figure 3**). This loop initiates with autoimmune-mediated DE, where dilated ducts accumulate retained secretions, exfoliated epithelial cells, and cellular debris—generating DAMPs. These DAMPs activate PRRs on mammary epithelial cells, including TLR4 and NLRP3 inflammasomes, triggering NF-κB signaling. NF-κB serves as a master regulator, driving transcription of pro-inflammatory cytokines (IL-1β, IL-6), adhesion molecules (ICAM-1) (28, 72), and core EMT transcription factors (Snail, TWIST) (73), thereby linking ductal damage to EMT initiation.

Once EMT is triggered, epithelial cells undergo phenotypic conversion to mesenchymal cells, acquiring a fibroblast-like phenotype (21). These EMT-derived fibroblasts become key effectors, secreting CXCL12 and TGF-β1 into the local microenvironment. CXCL12 binds to CXCR4 on adjacent epithelial cells, activating NF-κB and PI3K/Akt/mTOR pathways to perpetuate EMT (58, 74); concurrently, TGF-β1 synergizes with NF-κB via Smad3/4-p65 complexes to enhance EMT and stimulate ECM synthesis (51). This creates a feedforward cycle: EMT generates fibroblasts that secrete factors reinforcing EMT in neighboring epithelia, while ECM deposition further distorts ductal structures, exacerbating DE and DAMPs release.

Critical to maintaining this loop is the IL-6/JAK/STAT3 axis, which exerts dual roles: promoting plasma cell survival via Bcl-2 upregulation (sustaining the inflammatory infiltrate) and enhancing Snail expression to drive EMT in ductal epithelium (38). Additionally, IL-1β amplifies the loop through PI3K/Akt-mediated stabilization of EMT transcription factors and ECM synthesis (28), while ICAM-1 reinforces NF-κB activation via a positive feedback loop, ensuring persistent EMT induction (45). Together, these mechanisms—NF-κB and JAK/STAT3 as convergent signaling hubs, EMT-derived fibroblasts as paracrine effectors, and plasma cells as inflammatory amplifiers—establish the EMT-fibrosis loop as the hallmark of PCM’s chronicity.

### 4.2 Distinctive features of PCM compared to other mastitis

PCM’s pathogenic landscape differs markedly from other breast inflammatory conditions, particularly acute lactational mastitis and granulomatous lobular mastitis (GLM) (75, 76), underscoring its unique therapeutic challenges.

Acute bacterial mastitis, typically triggered by bacterial infection (e.g., Staphylococcus aureus), resolves with antimicrobial therapy as the inflammatory response abates once the pathogen is cleared (77). In contrast, PCM persists due to its autoimmune foundation—evidenced by elevated antinuclear antibodies and responsiveness to immunosuppressants—and the self-sustaining EMT-fibrosis loop, which is absent in acute infection-driven mastitis.

GLM is another form of non-puerperal mastitis, which pathological characteristic is the presence of noncaseating granulomas centered around breast lobules (78, 79). Recent studies have shown that the occurrence of GLM is closely related to *Corynebacterium parakroppenstedtii* (80). While both PCM and GLM involve chronic inflammation and fibrosis, their immune microenvironments and drivers of tissue remodeling diverge. GLM’s pathogenesis is tied to dysregulated innate immune responses to bacterial components, with granulocyte and macrophage-driven inflammation dominating (81). In PCM, by contrast, the IL-6/JAK/STAT3/plasma cell axis is central: plasma cells not only secrete IL-6 to fuel inflammation but also contribute to ECM remodeling via matrix-degrading enzymes, and activate Bcl-2 to promote their survival, thereby enhancing fibrosis (22, 38, 45). Furthermore, the EMT-fibrosis loop, which is tightly linked to ductal ectasia in PCM, has no clear counterpart in GLM, where lobular destruction rather than ductal dilation is the primary structural anomaly (82).

### 4.3 Unanswered questions and future directions

Despite advances in understanding PCM pathogenesis, critical knowledge gaps remain, offering avenues for future research.

First, the role of microbial involvement remains contentious. While PCM is classically defined as a sterile autoimmune disorder, supported by elevated autoantibodies and responsiveness to immunosuppression, emerging evidence suggests potential bacterial triggers. Research suggests that PCM may be an inflammatory disease associated with bacterial infection and the resulting immune response (2, 83). Upregulation of IFN-γ and IL-12A expression in inflammatory cells in the breast stroma of PCM patients indicates that the adaptive immune response associated with bacterial infection—the Th1 immune response—plays a role in this disease. Resolving this paradox requires longitudinal studies to determine whether initial bacterial exposure primes autoimmune amplification (rather than persistent infection) and to identify specific microbial species that may drive DE or EMT initiation.

Second, the temporal dynamics of molecular pathways across PCM stages are poorly characterized. Current studies provide static snapshots of inflammatory mediators and EMT markers, but the transition from early DE to overt fibrosis remains unclear. For example, while IL-6/JAK/STAT3 and NF-κB pathways are implicated in both inflammation and EMT, it is unclear whether these pathways are sequentially activated (e.g., inflammation preceding EMT) or concurrently engaged, and how their activity shifts with disease progression. Addressing this requires multi-omics analyses of patient samples stratified by clinical stage (DE, inflammatory infiltration, fibrosis), paired with imaging to correlate molecular changes with ductal remodeling.

Third, in PCM, the mechanisms of immune cells beyond plasma cells—such as macrophages, T cells, or myeloid-derived suppressor cells (MDSCs)—in regulating EMT is underexplored. Macrophages undergo polarization in PCM: M1 macrophages secrete pro-inflammatory cytokines IL-1β that may induce EMT, while M2 macrophages, enriched in fibrotic stages, could promote TGF-β1-mediated EMT via paracrine signaling (84). T cells also have diverse functions that could impact EMT in PCM. T helper 17 (Th17) cells, via IL-17 secretion, may amplify inflammation and ECM deposition, whereas regulatory T cells (Tregs) might constrain excessive EMT through immunosuppressive cytokines like IL-10 (85). MDSCs have been shown to mitigate PCM severity when NLRP3 is inhibited (29), but their direct interaction with epithelial cells undergoing EMT, or their influence on fibroblast activation, remains speculative. Future studies could use co-culture systems of patient-derived mammary epithelial cells and other immune cells, paired with cytokine neutralization assays, to validate direct regulation of EMT by these immune cells.

Finally, translating preclinical findings to human PCM is imperative. It is important to emphasize that while studies using murine or bovine models (systems with noted differences from human PCM) have provided valuable mechanistic clues—such as the role of TGF-β1/Smad signaling in EMT induction or CXCL12-mediated fibroblast activation—these findings must be interpreted with caution. Murine models often rely on exogenous cytokine administration (e.g., TGF-β1 or LPS injection) that does not recapitulate the spontaneous, autoimmune-driven ductal ectasia of human PCM. Thus, all mechanistic hypotheses derived from animal models require rigorous validation in human PCM tissues—via approaches such as immunohistochemical co-localization of signaling molecules, single-cell transcriptomic profiling of epithelial and immune cells, or ex vivo functional assays using patient-derived cells—to ensure their relevance for guiding clinical translation.

By integrating fragmented mechanistic observations into a unifying framework, this review clarifies PCM as a disease driven by the interplay of autoimmune ductal damage, immune dysregulation, and the EMT-fibrosis loop—a PCM-specific circuit that distinguishes it from other mastitis and explains its therapeutic recalcitrance. Highlighting NF-κB and JAK/STAT3 as convergent signaling hubs, ductal ectasia as a pathogenic trigger, and plasma cells as central mediators of chronicity, our framework resolves prior ambiguities in how inflammation and fibrosis are linked in PCM. Importantly, this structured understanding not only reinforces why current non-specific therapies fail but also directs future research toward filling critical gaps—from resolving bacterial-autoimmune crosstalk to validating preclinical findings in human tissues. Ultimately, this framework lays the groundwork for translating mechanistic insights into targeted strategies that disrupt the EMT-fibrosis loop, offering a path to address the unmet clinical need for effective, stage-specific PCM treatments.

## 5 Conclusion

This review synthesizes current evidence to delineate PCM as a distinct inflammatory disorder driven by the intricate interplay between autoimmune-mediated ductal damage, immune microenvironment dysregulation, and EMT-dependent fibrosis. A core pathogenic feature of PCM is a self-perpetuating circuit: ductal ectasia-generated DAMPs activate PRRs, including TLR4 and NLRP3, triggering NF-κB signaling to induce the release of pro-inflammatory cytokines IL-1β and IL-6 and the upregulation of adhesion molecules ICAM-1. Concurrently, IL-6/JAK/STAT3 signaling exerts dual effects—promoting plasma cell survival via Bcl-2 and driving EMT through the upregulation of Snail, while TGF-β1 and CXCL12 amplify fibrosis through PI3K/Akt/mTOR pathways. Together, these mechanisms form the “EMT-fibrosis loop,” a PCM-specific self-amplifying process where EMT-derived fibroblasts secrete CXCL12 and TGF-β1 to reactivate EMT in adjacent epithelia, sustaining the disease’s chronicity and therapeutic resistance.

These mechanistic insights hold critical implications for advancing PCM clinical management. Moving beyond current surgical interventions and the non-specific immunosuppressive strategies, targeting convergent signaling hubs (e.g., NF-κB, JAK/STAT3) or disrupting the “EMT-fibrosis loop” (e.g., via TGF-β1 neutralizing antibodies, CXCL12/CXCR4 antagonists) offers a more precise, mechanism-driven approach to mitigate fibrosis and reduce recurrence. Clinically, JAK inhibitors (e.g., tofacitinib) already approved for autoimmune diseases show promise in preclinical models by reducing IL-6/STAT3-driven EMT (38), while TLR4 or NLRP3 inhibitors could blunt the initial DAMP-induced inflammatory cascade that seeds EMT (29). Additionally, recognizing DE as a pathogenic trigger—rather than a passive structural anomaly—highlights the potential of early interventions to prevent the onset of irreversible fibrosis.

Future research must address key gaps to fully translate these insights into clinical practice: longitudinal studies to map the temporal dynamics of “EMT-fibrosis loop” activation across PCM stages (DE, inflammatory infiltration, fibrosis); validation of loop components in human PCM tissues using patient-derived organoids or single-cell transcriptomics; and clinical trials testing combination therapies that co-target inflammation (e.g., JAK/STAT3 inhibitors) and fibrosis (e.g., TGF-β1 blockers) to disrupt PCM’s chronic cycle. Ultimately, unraveling and targeting the “EMT-fibrosis loop” represents not only a paradigm shift in understanding PCM pathogenesis but also a transformative opportunity to develop therapies that alleviate the long-term suffering of PCM patients, offering hope for reduced recurrence, preserved breast anatomy, and improved quality of life.
